# Short-Term Prognosis Value of sST2 for an Unfavorable Outcome in Hypertensive Patients

**DOI:** 10.1155/2020/8143737

**Published:** 2020-02-06

**Authors:** Anca Daniela Farcaş, Mihaela Mocan, Florin Petru Anton, Mocan-Hognogi Larisa Diana, Roxana Mihaela Chiorescu, Mirela Anca Stoia, Camelia Larisa Vonica, Cerasela Mihaela Goidescu, Luminița Animarie Vida-Simiti

**Affiliations:** ^1^Department of Internal Medicine, “Iuliu Hatieganu” University of Medicine and Pharmacy, Cluj-Napoca, Romania; ^2^Department of Cardiology, Emergency Clinical County Hospital, Cluj-Napoca, Romania; ^3^Department of Internal Medicine, Emergency Clinical County Hospital, Cluj-Napoca, Romania; ^4^Diabetes, Nutrition, and Metabolic Diseases Clinic, Emergency Clinical County Hospital, Cluj-Napoca, Romania

## Abstract

**Background:**

sST2 represents a useful biomarker for the diagnosis and prognosis of patients with heart failure, but limited data is available on its role in patients with hypertension. The aim of this study is to evaluate the short-term prognosis value of sST2 for an unfavorable outcome in hypertensive patients.

**Methods:**

This was a prospective observational study which enrolled 80 patients with hypertension, who were followed for one year. All patients underwent clinical, laboratory (including sST2), and echocardiographic assessment at baseline. The patients were grouped according to the cardiovascular (CV) events reported during the follow-up: group A (with CV events) and group B (without CV events).

**Results:**

Overall, 59 CV events were reported during the follow-up period. Compared to group B, the patients in group A had significantly higher sST2 levels, a higher number of CV risk factors, and a higher left ventricle mass. Except for the diastolic dysfunction parameters, the echocardiographic findings were similar in the two groups. Patients in group A had a lower *E*/*A* ratio, larger deceleration time, and increased telediastolic pressure as quantified by the *E*/*E*/*p* = 0.006, Kaplan-Meier analysis).

**Conclusions:**

sST2 levels were correlated with the risk of adverse CV outcomes in hypertensive patients and may represent a useful prognostic marker in these patients.

## 1. Introduction

The receptor of suppression of tumorigenicity 2 (ST2) (also known as IL-1R4, DER4, Fit-1, or T1) is a type 1 transmembrane protein encoded by the *IL-1RL1* gene, and the symbol for ST2 is approved by the Human Gene Nomenclature Database [1]. The gene is located on chromosome 2.12. The protein product of the ST2 gene encodes three isoforms identified in human tissues: a released soluble form (sST2, acting as a decoy receptor for IL-33, inhibiting IL-33/ST2L signalling) which can be detected in human serum, a transmembrane receptor (ST2L or ST2) discovered to be IL-33 in 2005, and a variant of ST2 (ST2V) [2].

Serum levels of sST2 are increased in conditions of ventricular biomechanical overload such as acute myocardial infarction (AMI) and proved to be useful in predicting mortality and heart failure (HF) in these patients [3]. Moreover, sST2 levels predict an outcome in patients with HF, and a variation of sST2 concentration over time is associated with prognosis [4]. Several other studies showed a correlation between levels of sST2 and the severity of HF, the left ventricular (LV) systolic function associated with valvular diseases [5], renal impairment, and other biomarkers of cardiac dysfunction such as B-type natriuretic peptide and C-reactive protein [6, 7].

Such data suggest that measurement of serum levels of sST2 may provide insight into the hemodynamic burden of the myocardium and might be useful for the early detection of cardiac disease, both systolic and diastolic. Taking these into consideration, investigating sST2 in patients with high blood pressure (HBP) was a logical stepwise approach as HBP affects the myocardium leading to left ventricular hypertrophy (LVH), left ventricular diastolic dysfunction (LVDD), and heart failure with preserved ejection fraction (HFpEF) [8, 9]. Actually, hypertension is the most important risk factor for HFpEF, with 75% of HFpEF patients being hypertensive [10]. The diagnosis of different pathogenic stages leading to HFpEF in hypertensive patients is based on ECG, which has low sensitivity but high specificity. Echocardiography, on the other hand, even though it is a sensitive and specific tool, lacks great accessibility, requires qualified personnel, and has low accuracy in obese or respiratory patients [9]. In the light of these findings, the identification of biomarkers useful for the early diagnosis and for prognosis of LVH, LVDD, and HFpEF in hypertensive patients is imperative and the data regarding this subject are scarce.

Thus, the present study was aimed at analyzing the relationship between serum levels of sST2 and the presence of LVH and LVDD in hypertensive patients, assessing at the same time the potential short-term prognosis value off sST2 for cardiovascular (CV) events in these patients.

## 2. Patients and Methods

### 2.1. Study Population

80 hypertensive patients (mean age 54.7 ± 13.5 years; 47.5% men and 52.5% women) were enrolled in a prospective observational study and followed for 1 year. The diagnosis of HBP was established according to the recommendations of the ESH/ESC guidelines [11]: SBP ≥ 140 mmHg and/or DBP ≥ 90 mmHg or if patients received treatment for HBP. Patients with chronic inflammatory or acute infectious diseases, heart disease (ischemic, congenital, and valvular heart disease, myocarditis, severe acute or chronic heart failure, and acute coronary syndrome), or pulmonary disease (COPD, asthma, and sleep apnea) were excluded. Clinical examination was performed, and demographic data, risk factors, and previous medical history were collected for all patients. Upon enrolment, all patients signed an informed consent and respected national and international legislation regarding clinical studies [12, 13].

### 2.2. Study Protocol

At baseline, the patients were examined clinically and fasting blood was collected by venous puncture, in the morning, after a rest of 5-10 minutes. Serum was obtained by centrifuging the coagulated blood for 15 minutes at 1000 × *g* and stored at -20°C until ST2 measurement, performed with the soluble ST2/IL-1R4 (human) and ELISA Tecan Sunrise reader. For the ST2 assay, the analytical limit of detection (sensitivity) was 5 pg/mL, intra-assay coefficients of variation (%) were 4-6%, and interassay coefficients of variation (%) were 8-10%.

Echocardiography was performed by an experienced sonographer using the 4-2 MHz probe on a Philips Affiniti 50 machine and measured the parameters for cardiac remodeling and diastolic and systolic function. The dimensions of the walls and heart cavities were measured in the M-mode, and the ejection fraction was estimated using Simpson's biplane method. The LV mass was determined using the modified Devereaux's formula [11]: values > 95 g/m^2^ in women and >115 g/m^2^ in men were considered diagnostic for the presence of LV hypertrophy.

Diastolic function was assessed using mitral diastolic flow parameters (*A* wave, *E* wave, *E*/*A* ratio, *E*-wave DT, and IVRT) and mitral annular (septal and lateral) tissue Doppler—mean value of *e*′ (*e*′m) and *E*/*e*(m). Patients were followed for 1 year, and all CV events, such as hypertension emergencies, episodes of LV failure, and unstable angina, were registered. Hypertensive emergencies were defined as severe hypertension (≥180/110 mmHg) in patients presenting to the emergency department in whom there is no clinical evidence of acute organ damage, in accordance with ESC Guidelines [11]. LV failure was considered in the presence of cardiac failure clinical signs (more than 2 symptoms from the Framingham score) and echocardiographic signs of diastolic and/or systolic LV dysfunction as described above. The diagnosis of unstable angina was established in accordance with the current criteria of the ESC Guidelines [14]: retrosternal pain with characteristic angina (de novo, aggravated by effort, and occurring at an early stage after percutaneous or surgical myocardial revascularization), the presence of electrocardiographic changes, and the absence of a myocardial enzymatic reaction (CK, CK-MB, and troponin). The following ECG changes were considered diagnosis criteria for unstable angina: ST depression ≥ 0.05 mV in two or more contiguous leads, ST depression combined with transient ST elevation (not fulfilling the STEMI criteria mentioned above), and T-wave inversion [14]. The ST and/or T variability on different ECG was considered another important diagnosis criterion for unstable angina.

### 2.3. Statistical Analysis

Statistical analyses were performed with IBM® SPSS® package version 19 (IBM Corporation, Armonk, NY, USA). Normally distributed continuous variables were expressed as mean and standard deviation and compared using the Student test. Continuous variables with abnormal distribution and ordinal variables were compared using the Mann-Whitney *U* test. Categorical variables were expressed as numbers and percentages, and the group comparison was done using the *χ*^2^ test. Correlation coefficients were calculated by linear regression analysis, while multiple regression analysis was applied for analysis of the dependency between variables. *p* < 0.05 was considered statistically significant. The predictive capacity of ST2 was evaluated using computed areas under the receiver operating curve (AUC). A value of *p* < 0.05 was deemed significant; confidence intervals (CI) were calculated for *p* = 0.05 as the threshold.

## 3. Results

Baseline clinical and demographic characteristics of the 80 patients are shown in Table 1. 30% of the patients had grade 3 HBP and 45% had grade 2 HBP. In 68.75% of the patients, BP values were correctly controlled at the first examination, in accordance with the recommendations of the European guidelines [15]. Patients had several modifiable or nonmodifiable risk factors. Dyslipidaemia was found in 90% of the patients (hypercholesterolemia in 52.5% of them, mixed in 32.5% of them, and hypertriglyceridemia in 5% of them). 27.5% of the patients were smokers, and 51.25% of them had weight problems (27.5% were overweight and 25% obese). Diabetes was associated with HBP in 35% of cases. During the one-year study follow-up, 36 patients (45%) presented a total number of 59 CV events. Patients with CV events were included in group A (36 patients), and those without were included in group B (44 patients).

Table 1 shows the characteristics of the patients in the two groups. There was no significant difference regarding gender and age of the patients, but patients in group A had a higher proportion of stage 3 HBP with very high additional CV risk (e.g., 10-year cardiovascular risk categories using European guideline recommendations for hypertension [15]) and worse BP control rates than those in group B. In group A, we found more patients presenting dyslipidaemia (with hypercholesterolemia and mixed dyslipidaemia), smokers with higher BMI (overweight and obese), and diabetics compared with group B. Serum sST2 levels were higher in patients in group A compared to group B.

There were no significant differences between the two groups except for the parameters of diastolic dysfunction. Patients in group A had a lower *E*/*A* ratio, a longer *E*-wave DT, and significantly higher end-diastolic pressure (quantified by the *E*′/*E* ratio) (Table 2).

Admission sST2 levels were significantly correlated with CV events (*p* < 0.001).  Table 3 shows the univariate association between CV events and log-transformed sST2 and clinical or echocardiographic parameters. CV event risk increases with increasing sST2 levels and glycemia. 80.7% of CV event number variability is determined by the sST2 level and glycemia. Regression analysis showed that 80.7% of CV event number variability can be explained by a 10 ng/mL increase in sST2 level and a 10 mg/dL increase in glycemia.

Diastolic function parameters—*E*/*A* ratio and *E*/em ratio—and LV mass were correlated with the incidence and number of CV events. Multivariate analysis showed that sST2 and fasting glucose independently increased the risk of CV events over a period of 1 year of follow-up (Table 4).

**Figure 1:**
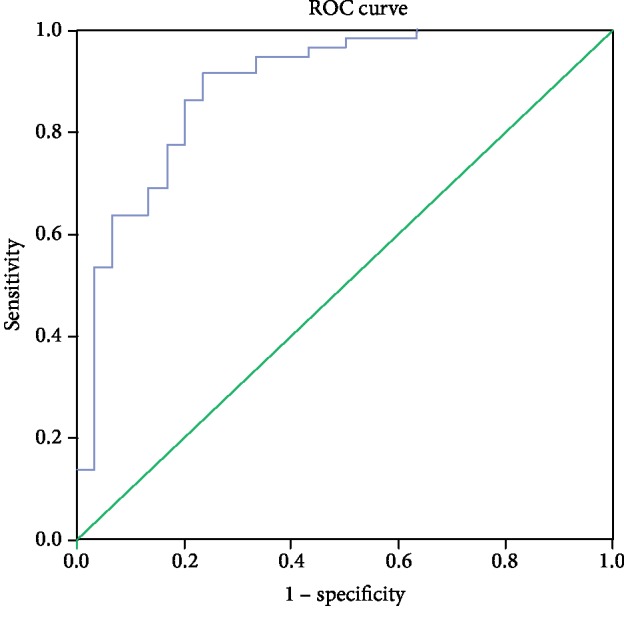
ROC analysis showing the sST2 sensitivity and specificity for predicting the CV events during one year after hospitalization.

**Figure 2:**
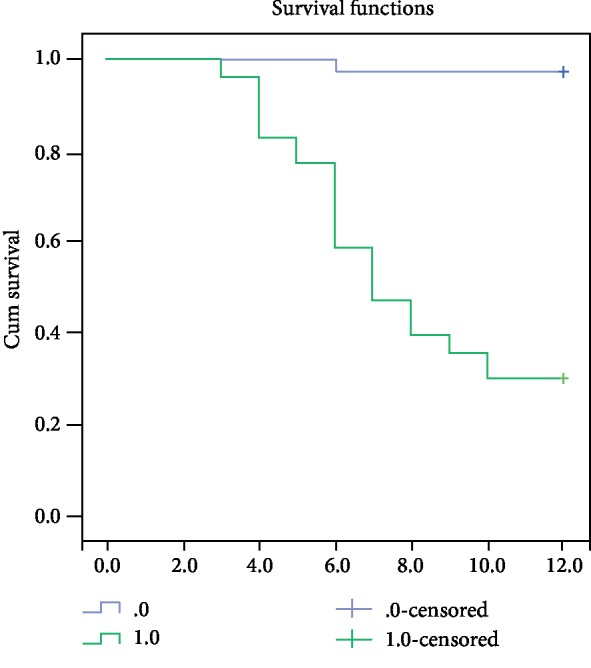
Kaplan-Meier curve analysis revealed that patients with sST2 > 28.5 ng/mL had a higher occurrence of CV events.

The receiver operating characteristic curve (ROC) for sST2 concentration identified 28.5 ng/mL as the optimal cut-off value to predict CV events with sensitivity and specificity of 94.4% and 69.1%, respectively (*p* = 0.000). An area under the ROC curve (AUC) was 0.84 (95% CI 0.78-0.89) which indicates the discriminative potential of this value of sST2 between high- and low-risk patients. Kaplan-Meier curve analysis showed that patients with sST2 > 28.5 ng/mL had a higher occurrence of CV events (HR 2.43, *p* < 0.001) (Figures 1 and 2).

## 4. Discussions

Our study showed that sST2 levels are higher in hypertensive patients with CV events than in those free of CV events. sST2 levels are correlated with higher LVM, a number of CV risk factors, and the presence of LVDD. Fasting glucose and sST2 are correlated with CV events on the short term.

### 4.1. sST2, HBP, and LVDD

Other small studies of sST2 variations were performed in hypertensive patients, with similar results. Ojji et al. in 2013 suggested a link between LV geometry and sST2. The authors raised the hypothesis that sST2 level was not only affected by hypertensive LVH but might be a future biomarker in differentiating concentric hypertrophy from normal geometry in HBP [16]. The study comprised 133 consecutive patients diagnosed with HBP, with 37% presenting LVH [16]. Later on, the same group showed that sST2 serum levels correlated strongly with clinical and echocardiographic parameters and correlated well with NT-pro-BNP [17]. So sST2 might be useful to distinguish between hypertensive patients with and without LVH. Our group (Farcas et al.) has previously shown that sST2 could be useful as an early diagnostic biomarker for cardiac remodeling and altered diastolic performance in HBP, providing additional data to echocardiography. It could represent a milestone in early detection of cardiac performance alteration [18]. Furthermore, Wang et al. performed a larger study on 344 patients with HBP and HFpEF and showed that sST2 measurement provides diagnostic aid of stable HFpEF, correlated with NYHA class and LVDD [19]. In these patients, combined measurement provided an advanced risk stratification value compared to one biomarker measurement alone [19]. As a physiopathological explanation of the link between altered diastolic performance and high sST2 levels in hypertensive patients, Bartunek et al. showed that in humans with cardiac hypertrophy and heart failure, serum sST2 correlates with the diastolic load and has an extramyocardial source [20]. Furthermore, Zile et al. discovered that HBP in the presence of HFpEF alters passive myocardial stiffness with simultaneous increase in inflammation and fibrosis biomarkers, such as sST2, sustaining the hypothesis that the development of HFpEF depends on changes in both collagen and titin homeostasis [21].

### 4.2. sST2 and Short-Term Outcomes

In our study, we found that sST2 correlated with short-term prognosis for CV events in hypertensive patients. Released as a consequence of myocardial strain, influenced by inflammation and imbalance of the extracellular matrix, sST2 may be a suitable biomarker for prognosis, i.e., LVDD progression to HF [22]. Most of the large studies focused on the prognosis value of sST2 in HFrEF [23–25] and in acute coronary syndromes [3], rather than in ambulatory patients. In a multicentric study of 447 HFpEF patients admitted for acute HF, the authors demonstrated a comparable prognostic value of sST2 in both HFpEF and HFrEF [26]. When evaluated in ambulatory patients with HF, sST2 provided “valuable long-term risk stratification information in HF beyond that reported by other biomarkers of stretch, inflammation, necrosis, and remodeling” [27].

### 4.3. sST2 Relation to Other Imaging Methods

One of the main findings in our study is the relations of sST2 levels and LVDD diagnosed by echocardiography. The potential diagnosis capacity of sST2 in LVDD and its correlations with echocardiographic findings were elegantly reviewed by DeFilippi et al. [28]. In brief, large studies such as the Cardiovascular Health Study, comprising older patients (>65 years old) with LVDD ≥ grade 1 present in 24.1%, showed that sST2 was strongly associated with LVDD (OR 1.35 (95% confidence interval 1.06-1.72)) and especially with echocardiographic criteria (*E*/*A* mitral inflow) and had the capacity to improve the diagnosis accuracy [29]. On the other hand, the Framingham study showed no correlation between sST2 and echocardiographic criteria for LVDD, supporting the idea that sST2 might be more suitable for a risk screening strategy in large cohorts, rather than a screening tool for structural heart disease [30]. Moreover, Daniels et al. evaluated 588 ambulatory patients with HF and found that sST2 was predominantly associated with right ventricle and not LV structural alterations [31]. The question remains whether this strong association between sST2 and LVDD in elders is a result of a cardiac-specific effect or it is influenced by general factors such as inflammation or vascular stiffness, which are common findings in aging patients [28].

### 4.4. sST2 in comparison to Other Biomarkers

Our study focused on single-marker evaluation, rather than on a multimarket strategy, for the diagnosis of LVDD. In our previous work, we assessed the potential of N-terminal pro-B-type natriuretic peptide as a diagnosis biomarker for LVDD in hypertensive patients with metabolic syndrome [32]. As in patients with metabolic syndrome, in our study group, 34% of the patients were diagnosed with diabetes mellitus (DM) and fasting glucose, along with higher sST2 levels, is associated with higher risk of CV events. In this respect, Ruocco et al. showed that ST2 was higher in patients with LVDD and DM, it significantly correlated with glycosylated haemoglobin (HbA1c), and it was related to an adverse event occurrence within 6 months and with poor prognosis [33]. Miller et al. have recently demonstrated that sST2 is related to DM and inflammation rather than CV risk factors, blood pressure, or smoking [34]. The pathogenic link between circulating sST2 and DM is still not clear but could be causal. The authors hypothesized that sST2 not only is a biomarker but also may contribute to the pathogenesis of diabetes via IL-33 interactions. The IL-33/ST2 pathway may participate in the inflammatory and remodeling processes of various tissues in patients with DM [34]. The potential diagnosis biomarkers in cardiac remodeling after myocardial infarction in patients with HBP and DM were a subject of a previous study published by our group. We showed that lower leptin levels were associated with reduced values of echocardiographic parameters of ventricular remodeling [35]. Further research should focus on sST2 and cardiac remodeling after myocardial infarction.

Other studies showed sST2 to improve discrimination when adjusted to multivariable models comprising N-terminal pro-B-type natriuretic peptide or galectin-3 [27]. sST2 as compared to other biomarkers has the advantage not to be influenced by confounders (renal function, BMI, or age), and its levels are modified by the progressing disease.

### 4.5. Limitations of the Study

There are some limitations to our study that are worth taking into consideration. First of all, our study is a cross-sectional study with a single measurement of biomarker levels and is limited by the small number of patients. As such, the study does not benefit from the inherent variability in time of the tested biomarkers. As outpatients were included, the study group consisted of rather young patients, equally distributed by sex. Thus, these results cannot necessarily be extrapolated to older populations. Also, the study population was not tested for underlying coronary artery disease, which could also present as LVDD. Moreover, the interaction between medical therapy and the serum levels of the studied biomarkers was not addressed in this analysis, which was more focused on the links between sST2 and echocardiographic parameters of LVDD. Finally, we have to acknowledge that the number of participants was relatively small because of the multiple exclusion criteria, but it was enough to give a study power of over 0.80. Larger clinical trials will be needed for the validation of an ST2-predictive value in clinical practice.

## 5. Conclusions

Serum levels of sST2 are strongly correlated with higher CV risk in hypertensive patients and have a predictive potential for poor prognosis in these patients. Fasting glucose and sST2 are correlated with CV events on the short term.

## Figures and Tables

**Table 1:** Baseline characteristics of all patients, group A, and group B.

Characteristics	All patients 80 (%)		Group A (HBP with CV events) 36 patients	Group B (HBP without CV events) 44 patients	*p*
Age	54.7 ± 13.5		55.23 ± 14.47	52.68 ± 11.53	0.06
Sex					
Men	38 (47.5%)	0.051	18 (50%)	20 (45.45%)	0.061
Women	42 (52.5%)		18 (50%)	24 (54.54%)	0.052
Hypertension (HBP)					
Stage I	20 (25%)	<0.001^a^	10 (27.77%)	10 (22.72%)	0.028
Stage II	34 (42.5%)	0.002^b^	12 (27.77%)	22 (50%)	0.001
Stage III	26 (32.5%)	0.023^c^	14 (38.88%)	12 (27.27%)	0.005
Additional cardiovascular risk					
Moderate	15 (18.75%)	0.002^a^	3 (8.34%)	12 (27.27%)	0.03
High	33 (41.25%)	0.023^b^	12 (33.3%)	21 (47.72%)	0.008^b^
Very high	21 (26.25%)	<0.001^c^		11 (25%)	0.000^c^
Controlled hypertension					
No	25 (31.25%)		21 (58.33%)	4 (9.09%)	<0.001
Yes	55 (68.75%)	<0.001	15 (41.66%)	40 (90.90%)	<0.001
Dyslipidaemia					
Without	8 (10%)		3 (8.33%)	5 (11.36%)	0.04
Hypercholesterolemia	42 (52.5%)		22 (61.11%)	20 (45.45%)	0.002
Mixed	26 (32.5%)		11 (30.55%)	15 (34.09%)	0.05
Hypertriglyceridemia	4 (5%)		—	4 (9.1%)	—
Smoke					
No	58 (72.5%)		18 (50%)	40 (90.9%)	0.001
Yes	22 (27.5%)	0.001	18 (50%)	4 (9.1%)	0.001
BMI					
Normal weight	32 (40%)	0.04^a^	5 (13.88%)	27 (61.63%)	0.003
Overweight	28 (35%)	0.45^b^	16 (44.44%)	12 (27.27%)	0.026
Obese	20 (25%)	0.03^c^	15 (41.66%)	5 (11.36%)	0.018
Diabetes mellitus (DM)					
No	54 (67.5%)		20 (55.55%)	34 (77.27%)	0.021
Yes	26 (32.5%)	0.001	16 (44.44%)	10 (22.72%)	<0.001

^a^Comparisons between 1 and 2; ^b^comparisons between 2 and 3; ^c^comparisons between 1 and 3.

**Table 2:** Clinical, laboratory, and echocardiographic data of patients in the two groups.

Variables	Group A (HBP with CV events) 36 patients	Group B (HBP without CV events) 44 patients	*p*
Age	55.23 ± 14.47	52.68 ± 11.53	0.06
Men	18 (50%)	20 (45.45%)	0.061
Women	18 (50%)	24 (54.54%)	0.052
SBP (mmHg)	156.00 ± 24.56	136.63 ± 26.71	0.023
DBP (mmHg)	95.22 ± 28.61	84.76 ± 12.92	0.024
BMI (kg/m^2^)	32.42 ± 6.71	27.84 ± 7.11	0.03
Uric acid (mg/dL)	6.75 ± 1.65	4.98 ± 2.13	0.079
Creatinine (mg/dL)	0.9 ± 0.51	0.79 ± 0.38	0.246
Serum glucose (mg/dL)	110.55 ± 31.64	104.36 ± 33.78	0.338
Total cholesterol (mg/dL)	160.72 ± 86.81	144.05 ± 58.84	0.57
HDL cholesterol (mg/dL)	47.05 ± 8.94	48.59 ± 13.46	0.48
LDL cholesterol (mg/dL)	104.81 ± 49	103.97 ± 52.88	0.49
Triglycerides (mg/dL)	128.70 ± 87.53	119.58 ± 83.52	0.08
ST2 (ng/mL)	52.71 (41.7–99.45)	21.34 (15.17–44.24)	0.002
Ascending aorta (mm)	30.92 ± 3.90	29.46 ± 8.45	0.002
Left atrium size (mm)	33.63 ± 9.56	34.04 ± 5.71	0.056
Left atrium area (mm)	10.65 ± 5.39	10.42 ± 6.92	0.053
End-systolic interventricular septum (mm)	12.19 ± 2.05	12.00 ± 2.26	0.051
End-diastolic interventricular septum (mm)	12.19 ± 2.05	12.00 ± 2.26	0.188
End-systolic LV posterior wall (mm)	13.07 ± 5.14	12.99 ± 5.344	0.051
End-diastolic LV posterior wall (mm)	11.68 ± 1.79	11.33 ± 2.64	0.052
End-systolic LV size (mm)	27.73 ± 12.29	27.04 ± 11.97	0.224
End-diastolic LV size (mm)	45.26 ± 6.04	42.36 ± 9.89	0.306
Right ventricle size (mm)	27.19 ± 7.08	25.14 ± 9.16	0.074
Stroke volume (mL)	34.83 ± 12.86	32.60 ± 11.70	0.001
LV mass (g/m^2^)	176.4 ± 26.2	155.13 ± 83.6	<0.001
LV mass index	146.95 ± 75.80	140.15 ± 82.93	<0.001
IVRT (ms)	112.12 ± 46.7	104.56 ± 57.03	0.009
*E*/*A*	0.78 ± 0.43	0.97 ± 0.59	0.0043
EDT (ms)	225.86 ± 75.06	204.64 ± 99.24	0.0032
*E*/*E*′m	10.64 ± 2.33	8.24 ± 3.56	0.0035

**Table 3:** Univariate and multivariate analyses showing association between CV events and log-transformed ST2.

	Univariate analysis		Multivariate analysis	
	Coefficient of correlation	*p*	Coefficient of correlation	*p*
ST2	*0.696*	<*0.001*	*0.64*	*0.002*
Fasting glucose	*0.380*	*0.020*	*0.34*	*0.0032*
LV mass (g/m^2^)	0.44	<*0.001*	0.38	0.051
*E*/*A*	0.28	0.027	0.34	0.07
*E*/*e*′m	0.31	0.021	0.45	0.06
TEj	0.289	0.006		

**Table 4:** Multivariate analysis showing sST2 and fasting glucose independent correlation with CV events on the short term.

Variable	Hazard ratio (95% CI)	*p*
	CV events	
sST2	2.43 (1.32–7.24)	0.005
Fasting glucose	1.43 (1.041–1.732)	0.0023

## Data Availability

The clinical data used to support the findings of this study are included within the article.
